# Evaluating Orientation Effects on the Fire Reaction Properties of Flax-Polypropylene Composites

**DOI:** 10.3390/polym13162586

**Published:** 2021-08-04

**Authors:** Swagata Dutta, Nam Kyeun Kim, Raj Das, Debes Bhattacharyya

**Affiliations:** 1 Institute of Appropriate Technology, Bangladesh University of Engineering and Technology (BUET), Dhaka 1000, Bangladesh; 2 Centre for Advanced Composite Materials, Department of Mechanical Engineering, University of Auckland, Auckland 1142, New Zealand; nam.kim@auckland.ac.nz (N.K.K.); d.bhattacharyya@auckland.ac.nz (D.B.); 3 Sir Lawrence Wackett Research Centre, School of Engineering, RMIT University, Melbourne, VIC 3000, Australia; raj.das@rmit.edu.au

**Keywords:** fire reaction properties, fire dynamic simulation, flax-polypropylene composites, orientation effect, fire behaviour

## Abstract

In this work, the fire reaction properties of flax-polypropylene (PP) composites were investigated at multiple sample angles both experimentally and numerically under two different heat flux conditions (35 and 50 kW/m^2^) in the cone calorimeter environment. An innovative testing setup which can accommodate a wide range of angles between 0° and 90° for the sample angle frame was developed to perform cone calorimeter tests at different sample angles. An advanced numerical predictive model based on the finite volume method was developed using the fire dynamics simulator (FDS) to quantify the dependency of ignition and combustion properties with sample angles. The numerical model was validated against experimental data from the cone calorimeter tests. The experimental and numerical analyses were conducted to quantify the effects of sample orientation on the different fire reaction properties i.e., ignition time, ignition temperature, burn time, heat release rate (HRR), critical heat flux, etc. The numerical method was utilised to analyse the mechanisms controlling the effect of heat convection and radiation blockage on the heating process. The study establishes that the sample orientation (with respect to the heat flux normal) has a significant influence on the fire reaction properties of natural fibre composites.

## 1. Introduction

Response of a material to an external heat flux and/or fire is quantified by the fire reaction properties. The properties include, but are not limited to ignitability, flame spread, heat release rate, flameout time and production of combustibles, such as smoke and gases which may be toxic, corrosive and obscuring. Among the fire reaction properties, heat release rate is the single most important property to the quantification of fire hazard [1]. Conventional polymeric composites (reinforced by synthetic fibres) have been widely used in different engineering applications due to their superior mechanical properties. However, due to their adverse environmental impact and lack of biodegradability, natural fibre reinforced composites are recognised as an alternative to their synthetic fibre counterparts because of their environmental merits.

High flammability of natural fibre reinforced composites is often critical in many applications. If a sufficient heat flux is applied, natural fibre composites begin to decompose and produce volatiles (small-molecular gases). The mixing of volatiles with ambient air produces a flammable mixture. Ignition occurs when the concentration of combustible gas rises up to the lower flammability limit [2]. Ignition is the primary step in the whole fire strategy and plays a critical role in fire growth. Understanding the mechanism of ignition is crucially important for predicting fire development and designing fire protecting standards. Over the past few years, a number of studies on the pyrolysis and ignition behaviour of different materials have been carried out [2,3,4,5,6,7,8,9,10,11,12,13,14,15,16,17,18,19,20,21]. However, most of the studies were focused on different grades of wood and Polymethyl methacrylate (PMMA) samples. Babrauskas [22] and Shields et al. [23] conducted separate studies on the effects of sample orientation on ignition and found similar results. The authors concluded that the ignition times for vertical orientation were longer than those of the horizontal ones. Shields et al. [23] also quantified the ignition times and reported vertical ignition times to be nearly three times longer than those of the horizontal ones. In contrast, Yang et al. [24] reported shorter ignition times for vertical orientation, although the authors used parallel silicon carbide rods as the radiation source as opposed to the conventional radiative heating elements. Tsai [11] conducted a comprehensive experimental work on the effect of orientation on different materials, such as PMMA, wooden products and foams. The study also reported longer ignition times for vertical samples; however, the study was qualitative in nature and lacked analytical interpretations. Dutta et al. [25] recently conducted a comprehensive study on the effects of vertical and horizontal orientations of natural fibre reinforced composites (NFRc) on their fire reaction properties. They developed a detailed numerical model for predicting the fire reaction properties of NFRCs and validated against experimental data with good agreement. Based on the findings of prior studies on the vertical and horizontal orientation effects, it is envisaged that a further study encompassing the complete spectrum of thermal load conditions that arise from different sample orientations would be highly valuable for understanding the response of real engineering structures and products subjected to various fire conditions [26].

**Figure 1:**
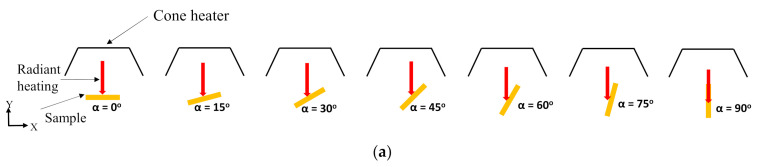

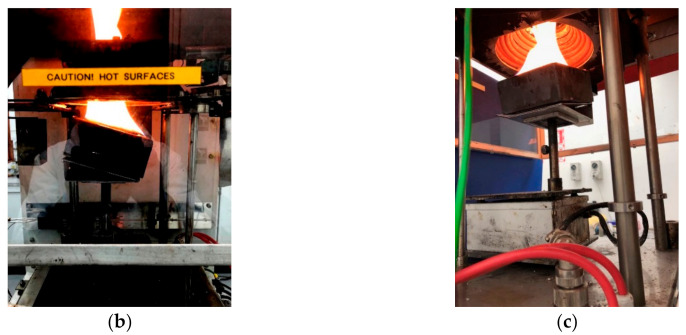
(**a**) Schematic 2D diagram of sample orientations relative to horizontal (i.e., heat flux normal) direction from the cone heater (**b**) Cone calorimeter experimental setup for an inclined sample orientation (15° orientation)—front view, and (**c**) side view.

In this research, a complete spectrum of sample angles (from 0° to 90°) with respect to the horizontal axis (*X*-axis, in Figure 1) was introduced to understand its effect on the ignition and combustion properties, in particular, for flax-polypropylene composites. The influence of sample angles on the ignition and combustion characteristics are reported for piloted ignition conditions. A computational fluid dynamics (CFD) model was also developed using fire dynamics simulator (FDS^®^) to analyse the mechanisms controlling the dependence of fire reaction properties with sample orientations under external heating. The implications of the study include but are not limited to the importance of angle of irradiance on the fire reaction properties which may help to better understand the behaviour of flax-polypropylene composites under extreme thermal loads. Moreover, the study aims at determining the influence of irradiance angle on the fire scenario, and whether the typical cone setups, namely the horizontal (0°) and vertical (90°) samples alone, can adequately characterise a fire situation.

## 2. Materials and Experiments

In this study, flax fibre and polypropylene (PP) were selected to prepare short fibre reinforced composites.

The composites were manufactured using 25 wt.% of flax fibres. Figure 1a elucidates the seven different orientations (α = 0°, 15°, 30°, 45°, 60°, 75°, 90°) of the sample exposed surface with respect to the horizontal direction (*X*-axis). The samples at each orientation were tested under two different heat fluxes (35 and 50 kW/m^2^). The cone heater was kept stable at its original position, emitting heat flux vertically downward, and the samples were oriented at different angles to understand the influence of the transient nature of the convective and radiative heating environment on the fire reaction properties. An innovative testing setup which can accommodate a wide range of angles between 0° and 90°, was developed to perform the cone calorimeter tests at different sample angles. In addition, the ASTM E1354 standard was carefully modified to accommodate all the sample angle frames in the cone calorimeter. However, the modifications were carried out only in terms of the distances from the centre of the bottom surface of the cone heater to the centre of the exposed surface of the samples. Notwithstanding, the distances were kept equal for all the simulation cases. Figure 1b,c shows the experimental setup used to perform the cone calorimeter test on 15° samples. The sample angle frame was positioned on top of the load cell and the sample holder on top of it in order to incline the exposed surface at an angle of 15° with respect to the horizontal axis (*X*-axis in Figure 1).

For the numerical modelling, physical and thermal properties of the material, such as density, thermal conductivity, specific heat capacity, emissivity, and heat of combustion, were specified according to the authors’ previous works in ref. [25,26]. The reference rate and temperature, heating rate, reaction order, and threshold temperature were used to define the pyrolysis reaction rate [27]. The present study utilised standard thermal (thermogravimetric analysis (TGA) and differential scanning calorimetry (DSC)) and physical characterisation methods (density measurement) to obtain accurate material properties. Furthermore, long flax (yarn) fibres and Moplen HP400L polypropylene (PP) were used for the flax fibre reinforced composites. The fibres were cut using a granulator to an average length of 2.9 mm. Later, the fibres and PP, were dried at 75 °C for 48 h and then physically mixed and extruded at the average processing temperature of 175 °C and at a screw speed of 170 rpm. In the next step, an injection moulding equipment with temperature profiles of 150/185/185/180 °C and an injection pressure of about 85 bar was used to produce the test samples. The fire reaction properties of the composite samples pre-conditioned at 23 °C and 50% humidity for 24 h were measured according to ASTM E1354 standard using a cone calorimeter (FTT Limited, East Grinstead, UK) at two heat fluxes (35 and 50 kW/m^2^). Details of the experimental procedure can be found in the authors’ other work [25]. The data reported in this study represent the averages of three repeat tests for each type of sample. For the convenience of data interpretation, the magnitudes of error bars were rounded off to whole numbers.

## 3. Numerical Modelling

**Figure 2:**
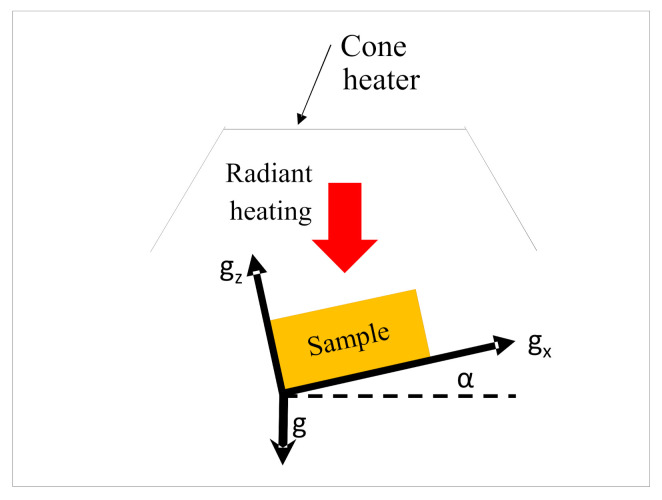
Schematic 2D diagram of the computational domain setup (not to scale).

The dependency of ignition and combustion properties with sample angles were assessed in terms of their ignition properties, such as ignition time and temperature, and combustion properties, such as heat release rate (HRR), peak heat release rate (PHRR), and burn time. A numerical predictive model based on the finite volume method was developed and implemented in FDS to understand the effects of convective heat transfer (due to flow of gases) on the pyrolysis and combustion characteristics of differently oriented samples. Details of the governing equations for the FDS simulation model can be found in [27]. In the simulations, the flax-PP composite was modelled as a homogeneous single layer although it consisted of several materials, namely flax and PP, in the analysis. Figure 2 shows the schematic diagram of the FDS domain of different models pertaining to different orientations (α = 0°, 15°, 30°, 45°, 60°, 75°, 90°) that were developed in the large eddy simulation (LES) domain in FDS. For each angle, the cone heater (radiation source) was kept at its original configuration and the sample’s exposed surfaces were changed each time to keep the exposed surface normal at the required angle to the radiation source. In all the cases, the computational domain was defined according to ASTM E-1354 standard; however, as stated before, the distance from the centre of the cone bottom surface to the centre of the exposed sample surface was changed according to the experimental setup (40 mm) and kept equal for all the orientations. The size of the computational domain was 400 × 400 × 500 mm and appropriate boundary conditions were applied to different components of the domain. Details of the boundary condition can be found in the authors’ earlier work [25]. The gravitational acceleration field orientation vector in FDS was altered to simulate the change of sample angles. It was reported in [28] that the change in the gravitational field vector in FDS can capture the effect of change in the sample orientations. The different gravitational orientation vector, **g** = (g_x_ g_y_ g_x_), with the components g_x_ and g_z_ are shown in Figure 2. The values of g_x_ and g_y_ are shown in Table 1, where g = −9.81 m/s^2^; g_x_ = gcos (α), g_y_ = 0, and g_z_ = gsin (α).

For piloted ignition condition, the igniter is modelled as a single grid region with a specified temperature (~1250 °C) and placed 20 mm above the sample exposed surface. The emissivity of the igniter is specified as 0.001 to minimise its influence on the radiant heating of the samples. The cone heater was constructed as a stair polygon shape.

In addition, two heat fluxes (35 and 50 kW/m^2^) were established by specifying a temperature (700 and 750 °C for 35 and 50 kW/m^2^, respectively) of the cone heater. The radiative heat flux on the surface was measured by placing heat flux gauges at three different locations on the exposed surface of the sample. A grid convergence study was conducted to systematically refine the grid to arrive at a converged grid that eliminates any effect of numerical discretisation on the prediction accuracy. Grid sizes of 4 and 3 mm were allocated into the horizontal and vertical directions, respectively. Details of the grid convergence study can be found in [16].

In all of the simulations, individual properties of the fibres and matrix were specified in the FDS input file. As illustrated in [27], FDS employs a ‘rule of mixture’ to calculate the effective thermal and physical properties of the entire composite material from the given properties of the constituents. In order to improve the computational efficiency, the number of transport equations to be solved was reduced by specifying ‘lumped’ configuration for the gas phase combustion products. The reaction fuels were composed of gas phase species, namely fibre and resin, and the chemical formulae of these constituents were also specified. For a particular material undergoing reaction, the combustion products or the yields for solid and gaseous products were specified according to the experimental observations. For the gas phase combustion to occur, sufficient thermal energy must be released to raise the cell temperature above the critical flame temperature. In this study, the flame temperature (ignition criteria) was set to the default value of 1600 K (1326.9 °C) for common hydrocarbons, as suggested by Beyler [29]. The flame suppression is related to the oxygen concentration and temperature. Specifically, the extinction of flame is related to the local oxygen mass concentration in the domain. In this work, the chemical formulae of the individual reaction fuels were provided to calculate the local oxygen mass concentration based on the state relationships (derived from the first principles of thermodynamics) between oxygen and fuel consumption during combustion. In the mixing-controlled combustion model, a state relationship typically refers to the relation between the mass fraction of each species and the mixture fraction. All the simulations were conducted by the ‘mixture-fraction combustion’ model in LES. The pyrolysis process in the solid phase was modelled using a single step global Arrhenius reaction. Moreover, the gas phase finite rate chemical reaction was modelled as a single step first order Arrhenius reaction. The chemical formulae of flax were defined as C_6_H_10_O_5_ as the user input to simplify the mixture-controlled combustion model in FDS. Suitable gas species for PP were specified as pre-defined FDS entities. There were few assumptions made during the simulation, viz., combustible gases are immediately available and do not accumulate within the material; thermal equilibrium is established between gases and solid material; no change of incident heat flux during expansion of heated surface; reaction rate kinetics obtained from TGA can be used to simulate the fire reaction properties.

## 4. Results and Discussion

### 4.1. Effects of Sample Orientation Angle (α) on the Time to Ignition (t_ig_)

The time to ignition (*t_ig_*) is defined as the duration from the point of first exposure to the incident heat flux to the instant when a visible flame is first observed. The temperature of the flammable mixture must be sufficiently high to initiate the gas-phase exothermic reactions. In this study, results of only piloted ignition are reported. However, at any given heat flux and sample angle, it is evident that the auto-ignition will be longer.

**Figure 3:**
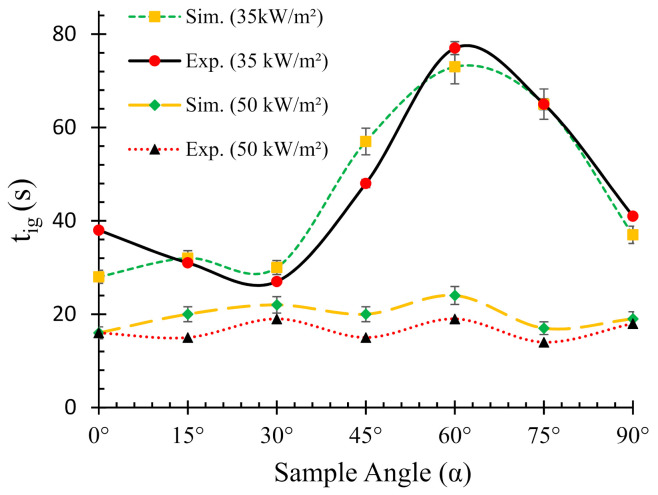
Relationships between ignition times (*t_ig_*) and sample orientation angles (*α*), H = horizontal (α = 0°), and V = vertical orientation (α = 90°).

Figure 3 elucidates both experimental and numerically predicted *t_ig_* for different sample orientations under two different heat fluxes. For both heat fluxes (35 and 50 kW/m^2^), the predicted times to ignition show good agreement with the experimental ones. In addition, for the lower heat flux case (35 kW/m^2^), an S-shaped curve was observed from both experimental and numerical results. On the other hand, a relatively linear curve with slight variations in *t_ig_* was obtained for the higher (50 kW/m^2^) heat flux case. Furthermore, the *t_ig_* for 35 kW/m^2^ was significantly higher than their higher heat flux counterpart. The lowest (at 28 s) and highest (at 73 s) *t_ig_* values for 35 kW/m^2^ flux was obtained for 0° and 60° sample angles, respectively. The difference in *t_ig_* between the 0° and 90° samples was merely 9 s as compared to 45 s between the 0° and 60° samples. On the contrary, the lowest (at 16 s) and highest (at 24 s) *t_ig_* values for 50 kW/m^2^ flux were obtained for 0° and 60° samples, respectively. However, it is important to note that the 90° samples (as shown in Figure 1), did not ignite when subjected to 35 kW/m^2^ heat flux; however, for the 50 kW/m^2^ heat flux, the samples ignited after a long time (~242 s) and were completely flamed out at 1065 s with a peak heat release rate of ~190 kW/m^2^. It was evident from the results that the intensity of the radiative environment at this angle was very low since only a small part (thickness direction) of the sample was exposed to the radiative normal. Hence, it was established that if the surface normal is perpendicular to the radiative source (90° samples as shown in Figure 1), the material exhibited very high ignition and flameout times posing no significant threat (fire hazard) to the surroundings. Hence, in the rest of the paper, for better comparative analysis, instead of the results for 90° samples (as shown in Figure 1), the cone calorimeter results from the standard vertical (90° (V)) orientation setup (according to ASTM E1354) were reported to compare against different sample angles. In this way, fire reaction properties of the intermediate sample angles (15–60°) can be compared with the typical cone calorimeter setups, viz. horizontal (0°) and vertical (90°) samples. A large difference in the *t_ig_* values for different angles was obtained for lower heat flux. In contrast, it was observed that the ignition time weakly depended on the angles for higher heat flux (50 kW/m^2^). This trend can be explained by the differences in the thermal environments, dominant heat transfer modes, fire plume directions and heat transfer modes of the different orientations. In addition, the convective heat transfer coefficient (*h*) is not the same for different sample orientations. This is one of the several reasons for which the decomposition behaviour differs in different orientations. It is also very difficult to determine the exact value of *h* at different orientations of the sample. However, it is roughly estimated according to the Nusselt number (*Nu* = *hL*/*k,* where *h* = convective heat transfer coefficient, *L* = length, *k* = thermal conductivity). Therefore, a difference in the convective flow can also affect the *t_ig_*. For 0° samples, the convective heat flow is established in the opposite direction of the radiative heat flux. On the other hand, for other different sample angles (15–60°), the convective flow changes direction from 0° (radiative heat flux direction) towards 90° (perpendicular to radiative heat flux) and gradually the flow occurs at 90° with respect to the radiative heat flux for vertical (90°) samples. Therefore, the convective heat loss being dependent on the orientation of the samples influences the intensity of the effective heat flux, i.e., the difference between the imposed radiative heat flux and convective heat flux loss, on the exposed surface. In both heat fluxes, the simulated curves slightly over-predicted the *t_ig_*. The result can be attributed to the higher pyrolysis rate and combustible gas flow in the numerical analysis rather than the average values of those occurring in the tests. In the FDS simulations, TGA results were used to determine the kinetic parameters. Therefore, the difference in the thermal environments between TGA and the cone calorimeter may also affect the pyrolysis rate of the material. Additionally, the material pyrolysis rate and the flow of combustible gases influence *t_ig_*. The *t_ig_* values for 0° and 90° samples were previously verified by both experimental and numerical findings in the authors’ earlier work described in [25]. In this study, similar results (in terms of *t_ig_* values) were obtained for both angles.

#### Determination of Critical Heat Flux, ($\overset{•}{q_{\begin{array}{l} cr \end{array}}}$)

A well-known expression to relate ignition time and external heat flux was established by Delichatsios [25] and is given in Equation (1).

(1) $$\frac{1}{\sqrt{t_{ig}}}=\frac{1}{\sqrt{\pi }}\frac{2\overset{•}{q_{in}}+a\overset{•}{q_{cr}}}{\sqrt{k\rho c}\left(T_{ig}-T_{o}\right)}$$

**Figure 4:**
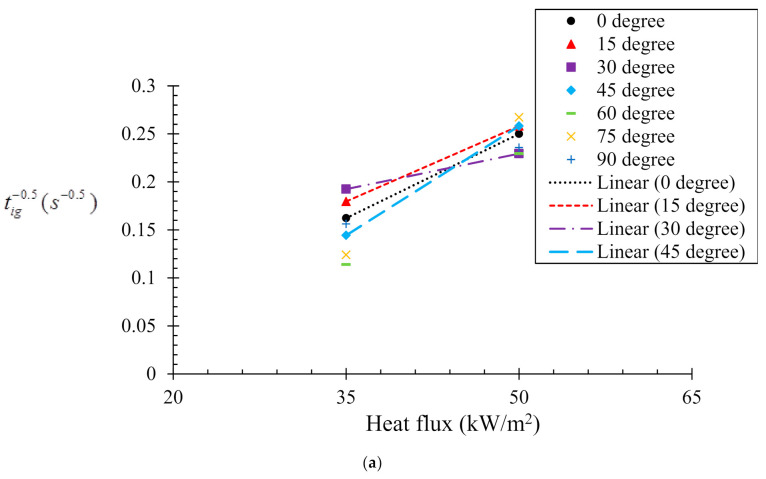

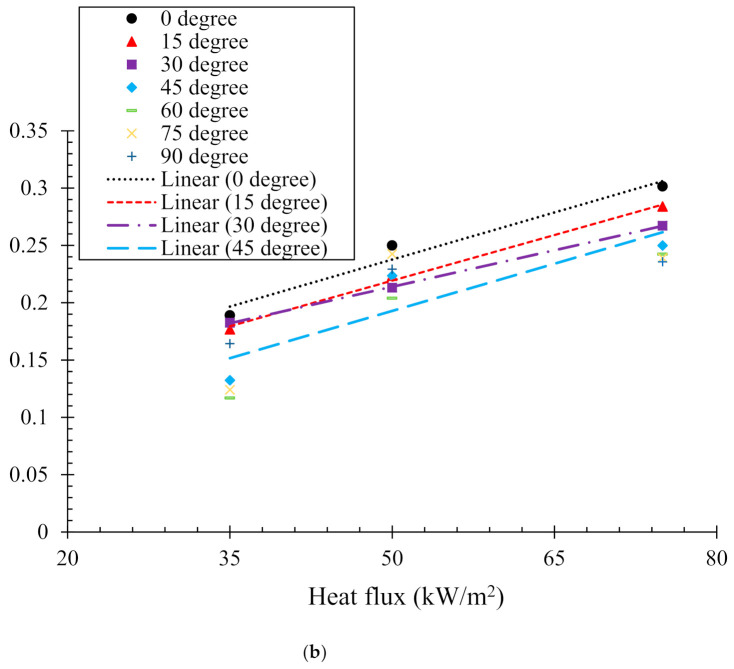
Relationships between $\sqrt{t_{ig}}$ and radiant heat flux according to Equation (1). (**a**) Experimental results and (**b**) numerical model results.

Here *t_ig_* is the time to ignition at a particular condition; *k*, ρ and *c* are the thermal conductivity, density, and specific heat capacity, respectively. *t_ig_* is the ignition temperature; *T_o_* is the initial temperature of the sample. Figure 4a,b illustrates the relation between $t_{ig}^{-0.5}$ and the incident heat flux obtained from experiment and numerical analyses, respectively. This shows the time to ignition is nearly inversely proportional to the square of the heat flux. In the case of the experimental analysis (Figure 4a), time to ignition values for two heat fluxes (35 and 50 kW/m^2^) were recorded. However, for the numerical analysis (Figure 4b), three different heat fluxes (35, 50 and 75 kW/m^2^) were used for evaluation of the numerical model prediction capabilities. The correlation between the ignition time and incident heat flux ($\overset{•}{q_{in}}$) can be obtained by linear fitting ($t_{ig}^{-0.5}$ = **a**$\overset{•}{q_{in}}$ **± b**). In general, the critical heat flux ($\overset{•}{q_{\begin{array}{l} cr \end{array}}}$) is considered as the lowest thermal load per unit area capable of ignition. Therefore, when $t_{ig}\to \infty$, the incident heat flux resembles the critical heat flux. The value of the critical heat flux can be obtained by extrapolating the line fits. The values of fitting constants *a*, *b* and critical heat flux ($\overset{•}{q_{\begin{array}{l} cr \end{array}}}$) obtained from both the experimental and numerical analyses are listed in Table 2. A close agreement (~5–10%) was found between the critical heat flux values obtained from the experimental and numerical analysis. It can be also noted that the critical heat flux increases with the sample orientation angle. However, after the sample orientation becomes equal to or larger than 60°, the ignition time showed insensitivity to the incident heat flux. The results of increasing critical heat fluxes reflect that the samples become harder to ignite with increasing sample angles. The range of critical heat fluxes obtained in this study is fairly close to that reported in [30,31]. The critical heat flux values obtained for the 0° and 45° samples are complemented by their *t_ig_* values (*t_ig_* becomes longer for 45° samples). In contrast, it was noted that for the 15° and 30° samples, although the critical heat fluxes were higher than that of the 0° sample, the *t_ig_* was smaller. This effect can be explained by the differences in the radiation heat flux blockage that occurs with increasing sample orientation angle. The radiation absorption coefficient used in the present simulation may have been over-estimated. Therefore, the effect of radiation attenuation of the incident heat flux caused by flowing pyrolysis gas is a vital parameter that influences the pyrolysis behaviour. The effect becomes more pronounced for higher incident heat fluxes. Another possible reason can be the mixing of volatiles with the air. For instance, the sample orientations can introduce strong discrepancy in buoyancy convection, the amount of mixing between air and volatiles and ability to flow out, which can contribute to the critical heat flux required for ignition.

For the horizontal case (0°), the radiation blockage is found to be higher. For the vertical case (90°), the pyrolysis gas flows upwards along the surface of the samples, therefore, causes smaller radiation attenuation compared the horizontal case.

### 4.2. Effects of Sample Orientation Angle (α) on the Ignition Temperature (T_ig_)

**Figure 5:**
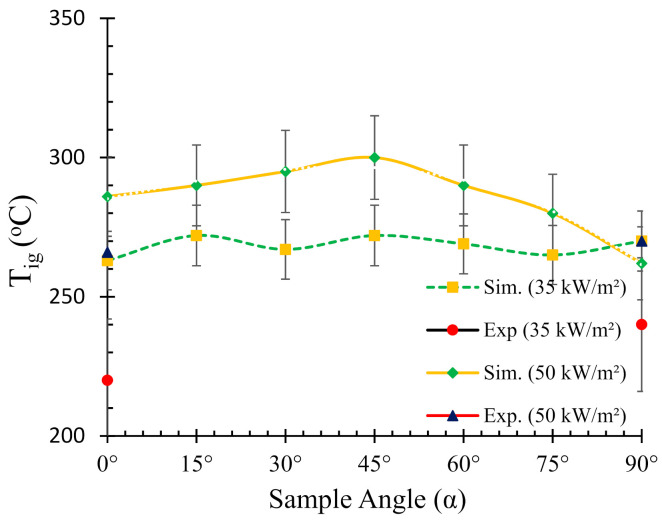
Relationship between the ignition temperature (*T_ig_*) and sample angle (*α*).

Experimental and numerically predicted ignition temperatures for various sample angles at two different heat fluxes (35 and 50 kW/m^2^) are presented in Figure 5. The experimental values of ignition temperature were obtained from authors’ previous work [25] for 0° and 90° (V) samples. As mentioned in Section 4.1, the fire reaction properties for 90° (V) samples are reported for standard vertical cone calorimeter setup. The reason for obtaining experimental *T_ig_* for only two angles was largely due to the constraint of modifying the sample angle frames for thermocouple insertion. The experimental values were meant for validation of the numerical simulations. However, ignition temperatures were obtained for all the angles in the numerical simulations to understand its variation with sample angle.

Notwithstanding, comparison of the experimental results for two angles with simulation can give an adequate insight about the prediction accuracy. It can be noticed from Figure 5 that the simulated ignition temperatures are significantly higher than those of the experimental ones. The trend from the simulated *T_ig_* curve for the 50 kW/m^2^ can be described by a gradual increase in *T_ig_* from 0° to 45° angles, followed by a steeper decrease from 45° to 90°. The maximum ignition temperature (~300 °C) was observed for 45° samples. From the experimental data, it was noticed that the ignition temperatures for the 0° and 90° samples were 266 and 270 °C, respectively. On the other hand, from the numerical simulation data, the ignition temperatures for the same sample orientations were 286 and 262 °C, respectively. The higher *T_ig_* values obtained in the numerical analysis can be explained by the accumulation of pyrolysis gases on top of the sample surface, absorbing a large amount of energy from the heater, followed by creating a hot and thick stagnation layer. Thus, the heat conduction is affected and the ability to penetrate and conduct heat flow through the stagnant layer possibly reduces which in turn dominates the net heat flux. In contrast, in the case of 35 kW/m^2^ heat flux, the *T_ig_* curve shows a relatively linear and constant trend with marginal variations in the ignition temperature with different sample angles. Therefore, it can be noted that the dependency of ignition temperature on sample angle is strongly related to the intensity of the radiative heat source. In an authors’ earlier work [25], a detailed description of the possible mechanisms responsible for the differences is provided.

In summary, it was identified that the consistency in the thermocouple readings can play a major role for the deviations. In addition, the ignition temperature was measured at the start of flame initiations, which is essentially based on flame visibility detection and thus slight deviations are expected. Furthermore, it was also concluded that several modelling assumptions are also pivotal to the variations in the predicted ignition temperatures. In the FDS model, it was assumed that the material would decompose by a simple one-step first order global reaction. Hence, the associated kinetic parameters of each composite component, namely the pre-exponential constant (A) and activation energy (E), are obtained from the major decomposition step for the simulation. The study also found if a temperature criterion was involved, the ignition temperature was largely related to the surface temperature, which had a counter-intuitive relationship with the activation energy. The ignition temperature was noted by visual observation of flame initiation in the Smoke View platform. The method is qualitative in nature, rendering some aberrations in the results. Given the wide range of input parameters associated with a full scale simulation as performed here, it is challenging to unwind the uncertainties pertaining to gas and solid phase routines. According to the authors’ knowledge, there has been no prior study conducted on the exact material systems for the full spectrum of angles as conducted here. However, Tzeng et al. [32] reported an ignition temperature range of 307–387 °C for timber materials under tested radiative heat fluxes from 21 to 36 kW/m^2^. Another work by Yang et al. [33] reported an ignition temperature range of 299–330 °C, under heat fluxes from 30 to 50 kW/m^2^.

### 4.3. Effects of Sample Orientation Angle (α) on the Heat Release Rate (HRR)

**Figure 6:**
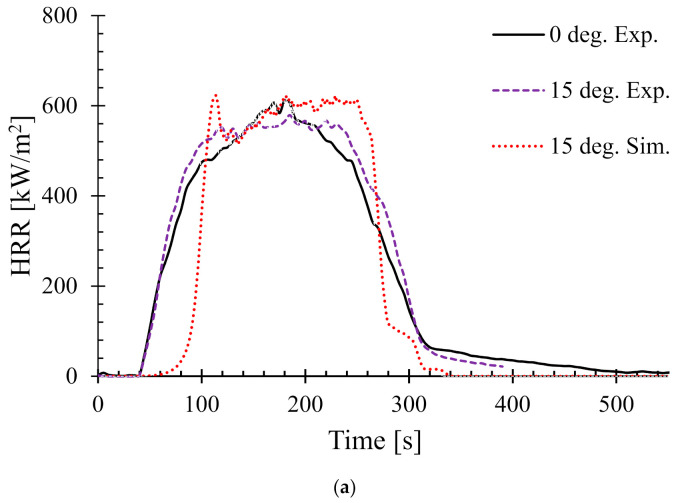

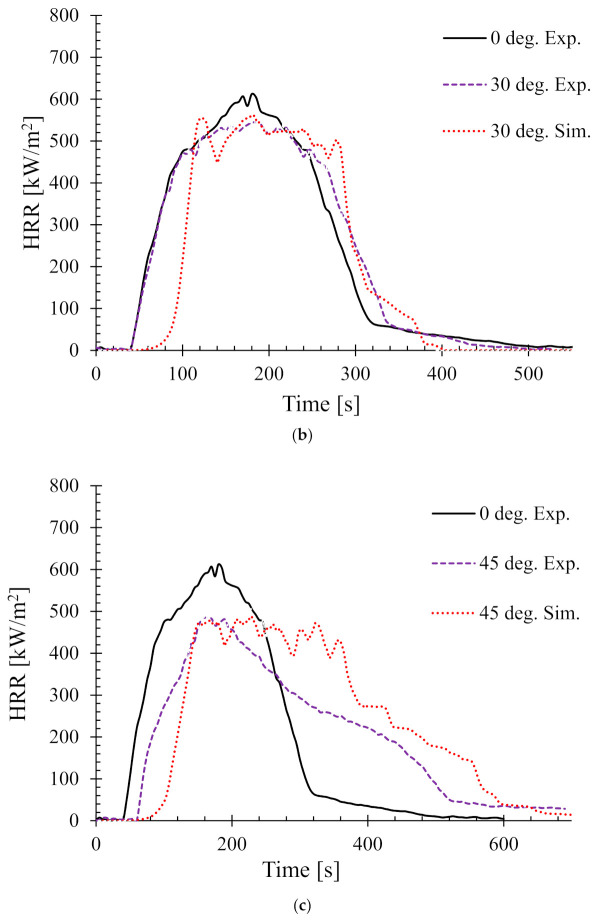

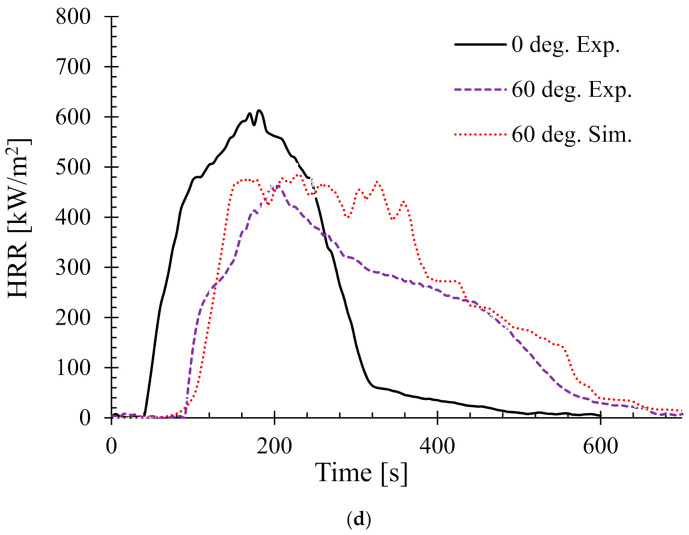
Heat release rates (HRR) for samples oriented at different angles at 35 kW/m^2^—(**a**) 15°, (**b**) 30°, (**c**) 45°, and (**d**) 60°.

Next the effect of sample angle on the heat release rate (HRR) was studied. Figure 6 shows the HRR of different sample angles at 35 kW/m^2^. The HRR for 0° and 90° (i.e., horizontal and typical vertical orientation) setups were validated by the authors’ earlier work in [25]. Therefore, in this work, only the samples orientated at 15°, 30°, 45°, and 60° are shown.

**Figure 7:**
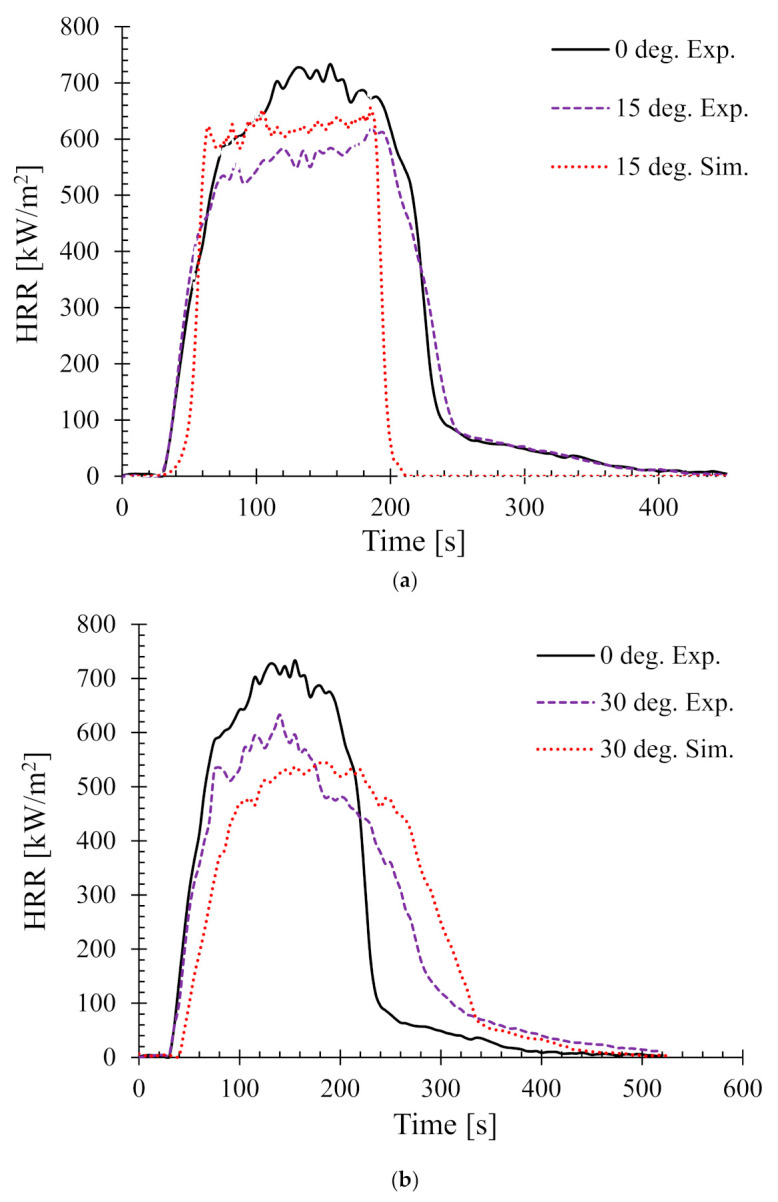

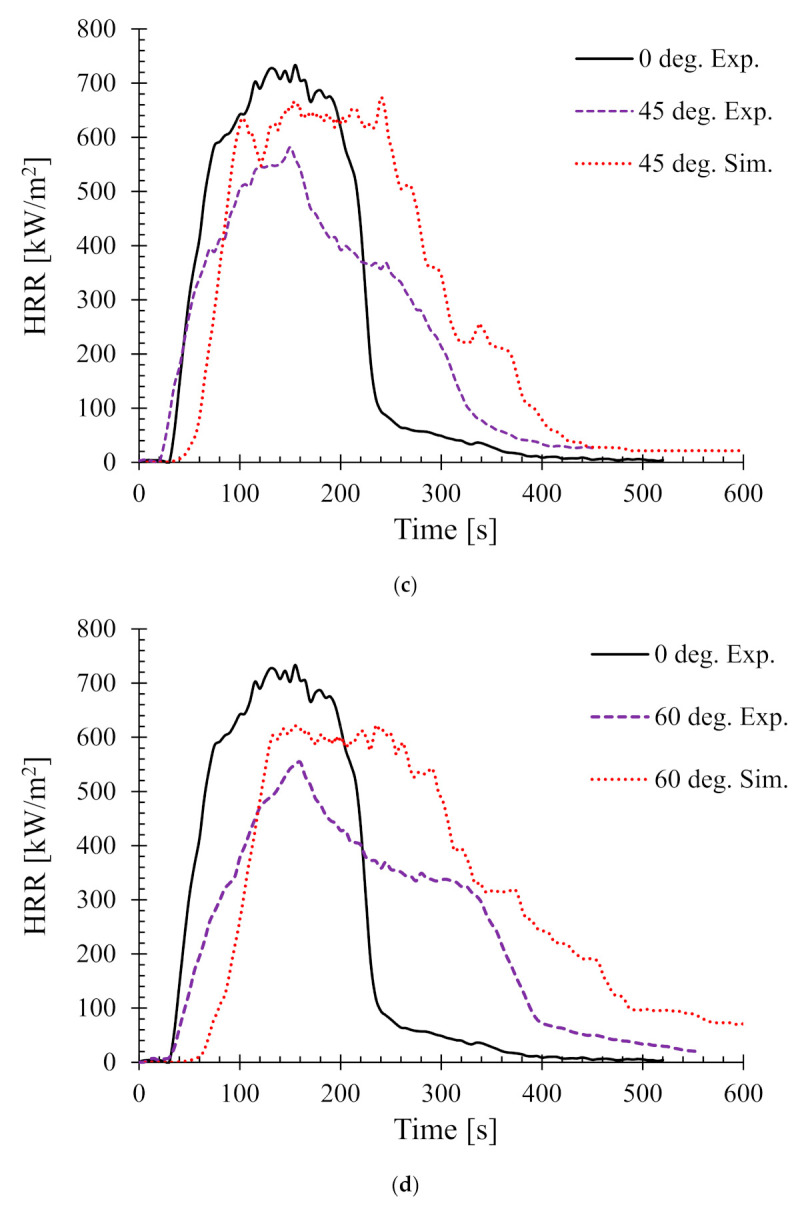
Heat release rates (HRR) for samples oriented at different angles at 50 kW/m^2^—(**a**) 15°, (**b**) 30°, (**c**) 45°, and (**d**) 60°.

**Figure 8:**
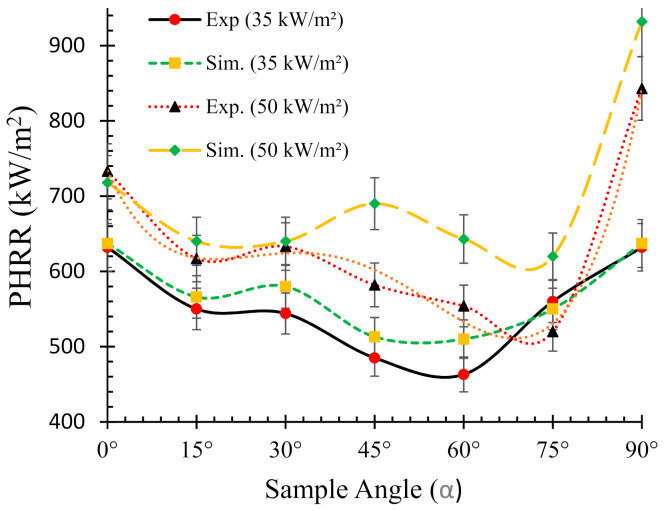
Relationships between the peak heat release rate (PHRR) and sample orientation angle (*α*).

In addition, as a benchmark and/or reference for comparison, the experimental HRR curves for 0° samples (horizontal orientation) are also overlaid in each of the figures. It can be seen that there is a distinct difference in the nature of the burning behaviour for different sample angles. For the 15° samples, Figure 6a shows that both the experimental and numerical results agreed very well and also show very little difference in terms of PHRR and total flameout time as compared to those of the experimental 0° samples. However, the predicted *t_ig_* for the same angle was slightly higher than that of the experimental ones. Moreover, for the 30° samples in Figure 6b, the experimental curves for both the 0° and 30° samples show marginal differences in terms of *t_ig_* and total flameout time. In contrast, the experimental and predicted PHRRs for 30° samples were less (~8%) than the 0° samples. Overall, the numerical model was able to predict the PHRR very accurately (within 2–4% tolerance). Furthermore, for both 45° and 60° samples shown in Figure 6c,d, respectively, the burning behaviour can be characterised by a higher *t_ig_* and total flameout time and a significantly lower (~18–20%) PHRR as compared to those of the 0° samples. Therefore, it can be concluded that the 45° and 60° samples burnt for longer period of time with less fire hazard (lower PHRR). Similarly, Figure 7 shows the HRRs for different sample angles at a heat flux of 50 kW/m^2^. It can be seen that the PHRR for all the sample angles was less compared to that of the experimental 0° samples. However, with the exception of the 15° samples, all other samples ignited later and burnt for a longer period of time as compared to the experimental 0° samples. The predicted PHRRs for all the sample angles were higher than those of the experimental ones. Figure 8 depicts the PHRR of flax (25 wt.%)–PP composites for various sample angles at two different heat fluxes. It can be seen that at a lower heat flux (35 kW/m^2^), the predicted PHRRs matched fairly accurately (~5–8%) with the experimental findings. The PHRR decreased quite significantly from 0° (637 kW/m^2^) to 60° (510 kW/m^2^) samples, then increased sharply to 650 kW/m^2^ (90° samples). For the higher heat flux (50 kW/m^2^), the numerical results and experimental data matched reasonably well for lower sample angles (from 0° to 30°). However, at higher sample angles, there were deviations between the predicted PHRR and the experimentally obtained one.

For instance, the predicted PHRR for 45° samples was 690 kW/m^2^, whereas the experimental value was 582 kW/m^2^. The effect of back surface boundary condition (insulated) in the numerical model may have contributed to the higher PHRR prediction. In addition, in FDS the mixing-controlled model using simple chemistry can only contain C, H, N and O as fuel molecules, and as a result, a higher mass loss of oxygen was calculated in the FDS model [29]. In general, the numerical model was able to predict the overall burning behaviour of the composite samples with reasonable accuracy.

### 4.4. Effects of Sample Angle on the Burn Time (t_b_)

**Figure 9:**
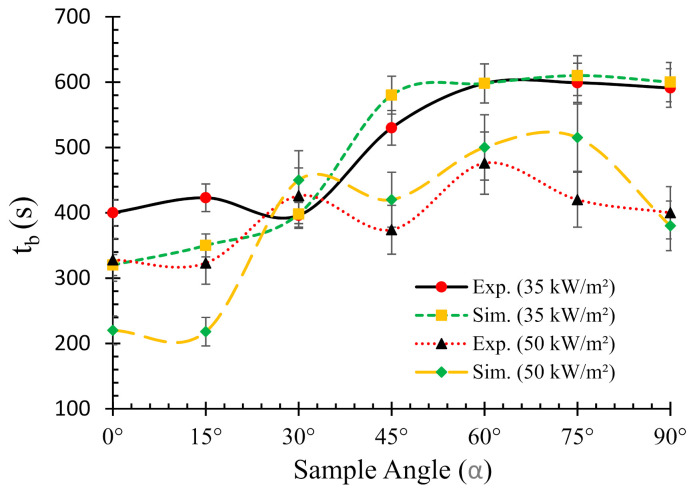
Relationships between the burn times (*t_b_*) and sample angles (*α*) for two different heat fluxes (0° = horizontal, 90° = vertical).

The burn time of a material is defined as the time it takes to complete the combustion process and/or burning after the onset of ignition. Figure 9 depicts the burn times of flax-PP composites obtained from both experimental and numerical results under two different heat fluxes. It can be seen from the figure that the burn times for 35 kW/m^2^ flux are greater in most of the cases as compared to their 50 kW/m^2^ flux counterpart.

However, no clear trend was observed. The data resemble a typical S-shape orientation with marginal variations. For all the samples, higher burn times were associated with the increase of sample angles. The only exception was 30° samples, where there was a sharp drop in *t_b_* and therefore, the lowest value (~396 s) among all the samples was attained for this case. Overall, the numerical model was able to predict the burn times for both heat fluxes with reasonable accuracy for most of the cases.

### 4.5. Fire Performance Index (FPI)

**Figure 10:**
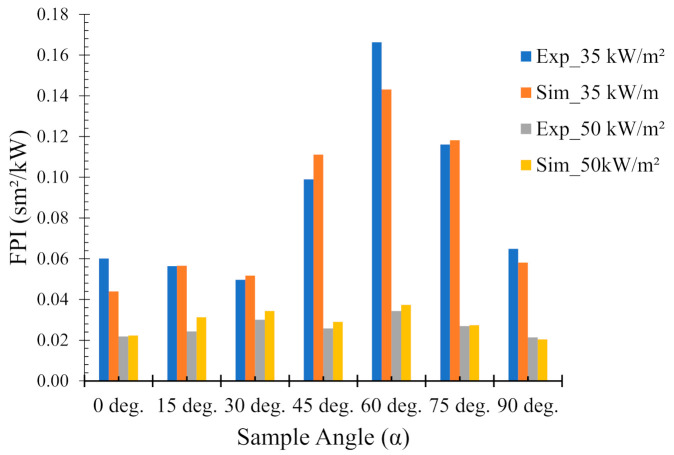
Comparison of experimental and numerical fire performance indices (FPI) of flax (25 wt.%)–PP composites at different sample orientation angles (0° = horizontal, 90° = vertical).

The fire performance index (FPI, unit: sm^2^/kW) is defined as the ratio of the time to ignition to the PHRR. The FPI was calculated in order to establish a ranking among the sample angles in terms of their fire hazard. Indeed, it is claimed in [34] that the higher the FPI, the better the flame-retardant performance. Figure 10 shows the FPI calculated from both experimental and numerical results for various sample orientations under two different heat fluxes. It is noteworthy that the numerical results show very accurate predictions for most of the sample angles. At a lower heat flux of 35 kW/m^2^, the FPI values are higher for all the sample angles as compared to their higher heat flux (50 kW/m^2^) counterparts. This is expected since at 35 kW/m^2^ the samples are exposed to lower fire intensities and/or temperatures, and hence have lower FPI values.

For both heat fluxes, the maximum value for the FPI (experimental and/or numerical) was obtained for 60° samples, signifying less fire hazard. In contrast, the minimum value for the FPI was observed for 0° and 30° samples for 35 and 50 kW/m^2^ heat fluxes, respectively. The results elucidate the importance of sample orientation in the evaluation of their performance to fire conditions.

### 4.6. Heat and Smoke Flow Temperature (T_s_)

**Figure 11:**
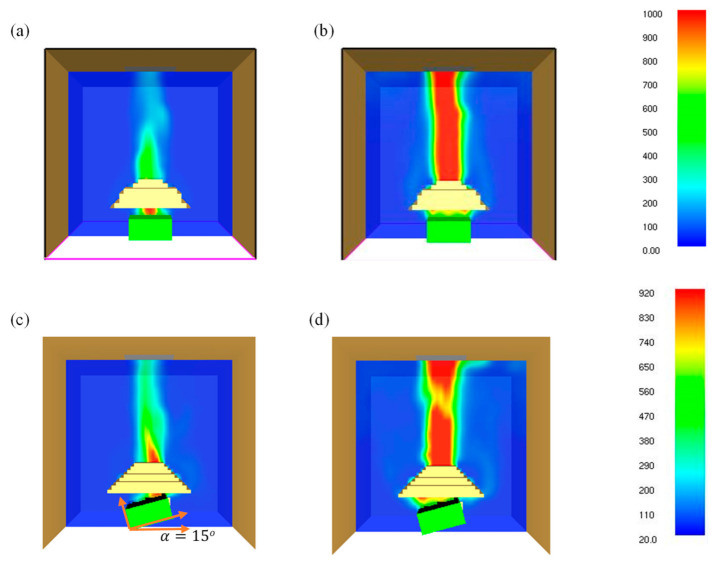
Effect of material orientation on the temperature of the exposed surface at 35 kW/m^2^ for (**a**) ignition phase—0° samples; (**b**) flashover (PHRR)—0° samples; (**c**) ignition phase—15° samples; (**d**) flashover (PHRR)—15° samples (temperature in colour bar).

The combustibles flow temperature (*T_s_*) is considered as one of the most intuitive parameters which can be used to measure the pyrolysis rate. Typically, the pyrolysis rate initiates at a relatively thin layer from the exposed end of the surface; therefore, the combustible flow temperature has a strong effect on the pyrolysis rate. The temperatures for 0° and 15° samples at the ignition and flashover phases at 35 kW/m^2^ are shown in Figure 11. It was found that for the 0° sample angle, the maximum surface temperature attained was 1000 °C as opposed to a lower value of 920 °C for 15° samples. In contrast, the maximum heat and smoke flow temperature for 90° samples was 770 °C. It is found that the surface temperatures are considerably different for different sample angles; therefore, the pyrolysis rate of the samples also varies accordingly. This results in a slower pyrolysis rate in solids and a lower fuel concentration in gases. However, the concentration of flammable mixture at the igniter location might also play a dominant role in the ignition process. For lower heat flux, the radiation blockage is considerably weak due to the production of less pyrolyzate. Moreover, radiation blockage appears to be highest for 0° samples and gradually decreases from 0° to 90°. This is due to the fact that as the sample angle increases, the pyrolysis gas flow tends to be higher along the surface of the samples (as the surface aligns more with the gravity direction), leading to smaller radiation attenuation. Chen et al. [28] conducted a study on thermally thick paulownia wood brick for three different orientations (upward, vertical, and downward) under heat fluxes ranging from 12 to 39 kW/m^2^. The authors also emphasised the influence of radiation blockage on the pyrolysis rate, ignition time and surface temperature.

The observation is similar to the findings of the present study. In the case of 50 kW/m^2^ heat flux, a similar trend is observed; however, as mentioned in Section 4.1, the time to ignition did not vary largely for different sample angles. This is possibly due to the less pronounced effect of radiation attenuation for higher heat fluxes. In addition, the surface temperature values were larger as compared to their lower heat flux counterparts.

## 5. Conclusions

A series of systematic experimental and numerical analyses were conducted to evaluate the effect of sample angle on the fire reaction properties of flax (25 wt.%)–PP composites subjected to 35 and 50 kW/m^2^ heat fluxes. The study reveals that the potential loss of net heat flux due to the convective loss to the ambience (radiative attenuation) can affect the resultant ignition time. It was shown that the critical heat flux increased with increasing sample angles. In addition, it was established from the study that if the surface normal of exposed surface is perpendicular to the radiative heat source, the material exhibited very high ignition and flameout times posing no significant threat (fire hazard) to the surroundings. The validation of the FDS model against experimental data reveals that the numerical model is able to predict the fire reaction properties (ignition and combustion) with a reasonable accuracy.

## Figures and Tables

**Table 1:** Values of different gravitational orientation vector components (in m/s^2^).

Orientation	0°	15°	30°	45°	60°	75°	90°
g_x_	0	−2.539	−4.905	−6.937	−8.496	−9.476	−9.81
g_z_	−9.81	−9.476	−8.496	−6.937	−4.905	−2.539	0

**Table 2:** Critical heat flux ($\overset{•}{q_{\begin{array}{l} cr \end{array}}}$) for ignition for different sample angles.

Angle (α)	Experiment			Simulation			$\overset{•}{q_{\begin{array}{l} cr \end{array}}}{\;}_{\left(\mathrm{kW}/m^{2}\right)}$	
	a	b	R^2^	a	b	R^2^	Exp.	Sim.
0°	0.0053	0.0463	0.99	0.0027	0.0249	0.96	8.75	9.25
15°	0.0052	0.0821	0.96	0.0027	0.0382	0.99	15.80	14.15
30°	0.0025	0.0542	0.91	0.0021	0.0359	0.99	21.70	17.12
45°	0.0076	0.1858	0.94	0.0027	0.0560	0.87	24.45	20.75

## Data Availability

Data shall be made available upon request.
